# Beneficial Effect of *Bupleurum* Polysaccharides on Autoimmune-Prone MRL-lpr Mice

**DOI:** 10.1155/2012/842928

**Published:** 2012-06-04

**Authors:** Yi-Wen Jiang, Hong Li, Yun-Yi Zhang, Wen Li, Yi-Fan Jiang, Ying-Ye Ou, Dao-Feng Chen

**Affiliations:** ^1^Department of Pharmacology, School of Pharmacy, Fudan University, 826 Zhangheng Road, Pudong District, Shanghai 201203, China; ^2^Department of Pharmacognosy, School of Pharmacy, Fudan University, 826 Zhangheng Road, Pudong District, Shanghai 201203, China

## Abstract

Systemic lupus erythematosus (SLE) is a chronic systemic autoimmune disease leading to inflammatory tissue damage in multiple organs. The crude polysaccharides (BPs) isolated from the roots of *Bupleurum smithii* var. *parvifolium* have anticomplementary activity and immunomodulatory functions on macrophages. To study its potential benefit on SLE, we examined effects of BPs on MRL-lpr mice, which have similar disease features to human SLE. MRL-lpr mice were treated orally with BPs 15, 30, or 60 mg kg^−1^ day^−1^ for 12 weeks and their SLE characteristics were evaluated. The results revealed that BPs elongated life span, improved kidney function, delayed lymphadenopathy, and reduced autoantibodies. It seemed to be mediated by inhibition of complement and macrophages activation and suppression of interferon-**γ** (IFN-**γ**) and interleukin-6 (IL-6) gene expression in the kidney. These results implicate that BPs may be an immunomodulator for the treatment of autoimmune diseases like SLE.

## 1. Introduction

Systemic lupus erythematosus (SLE) is an autoimmune disease involving inappropriate inflammatory responses, resulting in multiorgans dysfunctions like lymphadenopathy and glomerulonephritis. It is characterized by a polyclonal expansion of autoreactive lymphocytes and production of multiple autoantibodies, mostly in young females [1]. The main effectors of disease pathology are the diverse autoantibodies, immune complexes, complement activation, and autoreactive cells [2].

As an important link between the innate and adaptive immune system, monocytes/macrophages have been found to play an essential role in the pathogenesis of SLE [3]. Altered functions of these cells may play a dynamic role not only in the initiation of autoimmunity through abnormalities in phagocytosis but also in the perpetuation of the disease through abnormal signals such as increased costimulation of autoreactive T and B cells and in tissue damage [4].

MRL-Fas^lpr/lpr^ (MRL-lpr) mice with Fas mutation spontaneously develop an autoimmune disease similar to human SLE. Deposition of immune complex in the kidney triggers the production of proinflammatory mediators, resulting in macrophage and lymphocyte infiltration, and ultimately to glomerulosclerosis with renal failure in these mice [5]. Aberrant macrophage activities have been shown in numerous studies in MRL-lpr mice [6–8]. Indeed, MRL-lpr mice provide a more attractive model because their syndrome is rapid, spontaneous, and predictable. Because of this, we used MRL-lpr mice to attempt to evaluate therapies.

Radix Bupleuri (dried roots of *Bupleurum chinense* or *Bupleurum scorzonerifolium*), known as Chai-Hu, is one of the most frequently prescribed crude herbs in the prescriptions of traditional Chinese medicine for the treatment of inflammatory diseases [9] and autoimmune diseases [10]. Our previous experiments confirmed that crude polysaccharide isolated from *Bupleurum smithii* var.* parvifolium* (BPs) showed inhibitory properties toward complement activation [11] and had potent immunomodulatory activity on macrophages [12]. It had also been proved that BPs have beneficial effect on autoimmune disease induced by *Campylobacter jejuni* in BALB/c mice via inhibiting humoral immune hyperfunction and alleviating the activation of complement [11]. The present work is to study the effect of BPs on MRL-lpr mice and to learn its possible mechanism.

## 2. Materials and Methods

### 2.1. Isolation and Characterization of *Bupleurum* Polysaccharides (BPs)

The roots of *Bupleurum smithii *var.* parvifolium* were purchased from Shanghai Hua-Yu Chinese Materia Medica Co. Ltd and its identity was verified by Professor Shenli Pan at Fudan University. A voucher specimen (DFC-CH-H2003121602) of the plant material has been deposited in the Herbarium of Materia Medica, Department of Pharmacognosy, School of Pharmacy, Fudan University, Shanghai, China. The isolation and chromatographic studies of the crude polysaccharides from *Bupleurum smithii* var. *parvifolium* were completed as previously described [11].

### 2.2. Mice and Experimental Protocol

Eight-week-old female MRL-lpr and BALB/c mice were obtained from Slaccas-Shanghai Lab Animal Ltd. (SPF II Certificate; number SCXK2007–2005) and kept under specific pathogen free and normal housing conditions in a 12-hour light and dark cycle. All experimental protocols described in this study were approved by the Animal Ethical Committee of School of Pharmacy, Fudan University.

BPs and prednisone were ground and suspended in normal saline for administration, respectively. In the longitudinal study, twelve-week-old BALB/c mice were orally received normal saline as the control, and twelve-week-old MRL-lpr mice were orally received normal saline, BPs 60, 30, and 15 mg·kg^−1^·day^−1^, or prednisone 5 mg·kg^−1^·day^−1^ for 12 weeks.

During the physical exam, each mouse was palpated to determine the extent of lymph node enlargement that was present. Lymph node enlargement was scored as follows: 0, none; 1, mild enlargement (palpable, but not easily visible); 2, moderate enlargement (easily visible, but not interfering with mobility); and 3, severe enlargement (easily visible and interfering with mobility regardless of extent of interference) [13].

Urine was collected over 24 h in metabolic cages and stored at −80°C at week 24. Mice were sacrificed at the end of week 24 of age and serum was stored −80°C until measurement of antinuclear antibodies. Lymph node, spleen, thymus, and kidneys were removed promptly and one kidney from each mouse was stored into 10% formaldehyde before further analysis. Remaining kidneys were snap frozen in liquid nitrogen prior to storage at −80°C. The index of lymph node, spleen, or thymus was expressed as the ratio of lymph node, spleen, and thymus wet weight (g) versus body weight (g) (100×).

### 2.3. Immunoassay of Antibodies

Enzyme-linked immunosorbent assay (ELISA) was carried out for the detection of specific antibodies in sera of MRL-lpr and BALB/c mice (control group). For the detection of anti-dsDNA antibodies and anti-ssDNA antibodies, 96-well plates (Costar, Corning, NY) were coated with calf thymus DNA (Sigma) or denatured calf thymus at 50 *μ*g/mL. 10 *μ*g/mL histone from calf thymus (Sigma) was used for detection of antihistone antibodies. Murine serum was diluted at 1 : 200 in phosphate-buffered saline and horseradish-peroxide (HRP-) conjugated goat anti-mouse IgG antibodies were diluted at 1 : 1000 (Sino-American Biotechnology Company, Shanghai, China). Optical density (OD) was monitored at 492 nm using a well scanner ELISA reader (Multiskan FC, Thermo scientific). Results were indicated in Enzyme Index (EI). EI = 100 × OD_tested_  /(Mean OD_control group_ + 3SD) [14].

For the detection of total IgG, 96-well plates were coated with goat anti-mouse IgG (Wuhan Boster Biological Technology, Ltd.) 10 *μ*g/mL, 100 *μ*L/well. Sera were diluted at 1 : 10,000, 100 *μ*L/well. HRP-conjugated goat anti-mouse IgG antibodies were added at a 1 : 5,000 dilutions. The mice IgG standard (Sino-American Biotechnology Company, Shanghai, China) was used for standard curve fitting and immunoglobulin concentration calculating.

### 2.4. Assessment of Creatinine and Urinary Protein

Mouse creatinine was estimated in serum samples using a Creatinine Jaffe method kit (FengHui Medical Technology Company, Shanghai, China). Proteinuria was measured by Coomassie brilliant blue test [11]. Albumin (bovine serum) was used to make standard curves. Murine urine was centrifuged at 1000 × g for 10 min. The supernatant was diluted at 1 : 3 in normal saline. The optical density was measured at 540 nm after addition of Coomassie brilliant blue solution.

### 2.5. Hematoxylin and Eosin (H and E) Staining and Immunohistochemistry

The kidney was fixed with 10% formaldehyde and embedded in paraffin. Sections of 5 *μ*m thick were cut and stained with hematoxylin and eosin (H and E). Glomerular injury was blindly semiquantified by a renal pathologist. Sections were graded as follows: 0, normal; 1, a small increase of cells in the glomerular mesangium; 2, a larger number of cells in the mesangium; 3, complex endocapillary hypercellularity sometimes with mild sclerosis or necrosis; and 4, glomerular crescent formation, sclerosis, tubular atrophy and casts [15]. Usually, there were different grade lesions observed in a kidney; the most severe alteration was referred to as the grade of each mouse kidney and was taken into analysis.

For the detection of IgG deposits, the 5 *μ*m sections were deparaffinized, rehydrated, and incubated with peroxidase-conjugated goat anti-mouse IgG (Sino-American Biotechnology Company, Shanghai, China). Staining was visualized using chromogenic substrate solution 3-3′ diaminobenzidine (DAB).

### 2.6. Real-Time PCR

Total RNA was extracted from snap-frozen kidneys using Trizol reagent (Invitrogen, USA), and cDNA was synthesized from 2 ug total RNA by using random hexamers and SuperScript II Reverse Transcriptase (Invitrogen). SYBR Green I Dye detection system was used for quantitative PCR (qPCR) on Step One Plus Real-Time PCR System (Applied Biosystems, USA). 2 uL cDNA was amplified in a 20 uL PCR reaction system using recombinant Taq DNA polymerase (TAKARA, Japan). All reactions were performed in triplicate, and negative controls contained no template DNA. We used GAPDH RNA as an endogenous control for normalization. cDNA was subjected to 2-step PCR method: 95°C for 2 min; 40 PCR cycles (94°C for 10 s, 59°C for 10 s, and 72°C for 40 s) to detect MCP-1, IFN-*γ*, and IL-6 or subjected to 40 PCR cycles (94°C for 10 s, 57°C for 10 s, and 72°C for 40 s) to detect MHC-II, and subjected to 40 PCR cycles (94°C for 10 s, 57°C for 10 s, and 72°C for 40 s) to detect GAPDH. To verify that the primer pair produced only a single product, a dissociation protocol was added after thermocycling, determining dissociation of the PCR products from 60 to 95°C. Data were analyzed using the comparative threshold cycle (ΔΔCt) method.

### 2.7. Western Blotting

Kidney tissues of mice (100 mg) were separated by 12% SDS-PAGE under reducing conditions and transferred to PVDF membranes (Milipoler). Membranes were blocked with Superblock overnight at room temperature. Blots were probed with 1 : 200 dilution of primary antibody for C3, F4/80, IFN-*γ*, IL-6, MCP-1, MHC-II, and GAPDH (Santa Cruz, USA) for 2 hours in 5% milk/TBST. Membranes were next incubated with 1 : 2,000 dilution peroxidase-labeled second antibody (Santa Cruz, USA) for 2 hours. All membranes were visualized using DAB buffer. Densitometric analysis of the film was performed using a Model GS-2008 imaging densitometer (TANON) and analyzed using TANON analysis software.

### 2.8. Statistical Analysis

Quantitative variables were expressed as means ± SD. One-way analysis of variance (ANOVA) was used. If any significant change was found, post hoc comparisons were performed using Fisher's PLSD. Nonparametric data were analyzed by the Mann-Whitney *U*-test. *P*  value < 0.05 was considered significant.

## 3. Results

### 3.1. BPs Decreased Mortality Percentage and Lymphadenopathy of MRL-lpr Mice

**Figure 1:**
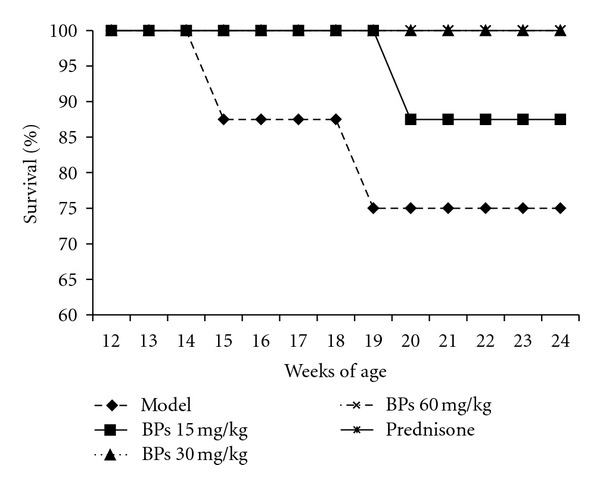
Effect of BPs on survival rate of MRL-lpr mice. MRL-lpr mice were grouped randomly and treated with BPs 15, 30, and 60 mg·kg^−1^·day^−1^, prednisone 5 mg·kg^−1^·day^−1^, or model from week 12 to week 24 (*n* = 8 per group); were data expressed as means ± SD.

The effect of BPs on survival was estimated by comparing the BP-treated group with vehicle-treated model group. After treatment for 12 weeks, the survival percentage of MRL-lpr mice (24-week-old) increased in BP- 30 mg·kg^−1^·day^−1^- (100%), BP- 60 mg·kg^−1^·day^−1^- (100%) and Prednisone- (100%) treated groups (Figure 1).

The lymphadenopathy of MRL-lpr mice was graded as lymph node enlargement. There was no significant difference in lymphadenopathy among five groups at 16 weeks of age. BPs 60 mg·kg^−1^·day^−1^ significantly delayed the lymphadenopathy after 8 weeks of treatment and prednisone after 9 weeks of treatment (*P* < 0.05) (Table 1).

### 3.2. BPs Decreased Organ Index of MRL-lpr Mice

**Figure 2:**
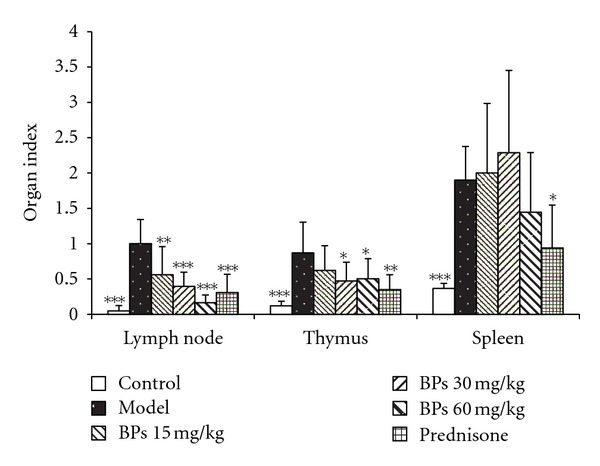
Effect of BPs on the index of lymph node, thymus, and spleen in BALB/c and MRL-lpr mice. MRL-lpr mice were grouped randomly and treated with BPs 15, 30, and 60 mg·kg^−1^·day^−1^, prednisone 5 mg·kg^−1^·day^−1^, or model from week 12 to week 24; data were expressed as means ± SD (*n* = 6–8); *P < 0.05, **P < 0.01; ***P < 0.001 compared with vehicle treated-model group, tested by ANOVA and Fisher's PLSD.

The index of lymph node, thymus, and spleen increased significantly in vehicle-treated model group when compared with control group. BPs inhibited lymph node swelling (*P* < 0.01), administration of 30 and 60 mg·kg^−1^·day^−1^ BPs inhibited thymus swelling (*P* < 0.05), while prednisone treatment significantly decreased the index of lymph node, thymus, and spleen (*P* < 0.05) (Figure 2).

### 3.3. BPs Reduced Autoantibody and Total IgG Levels in MRL-lpr Mice

**Figure 3:**
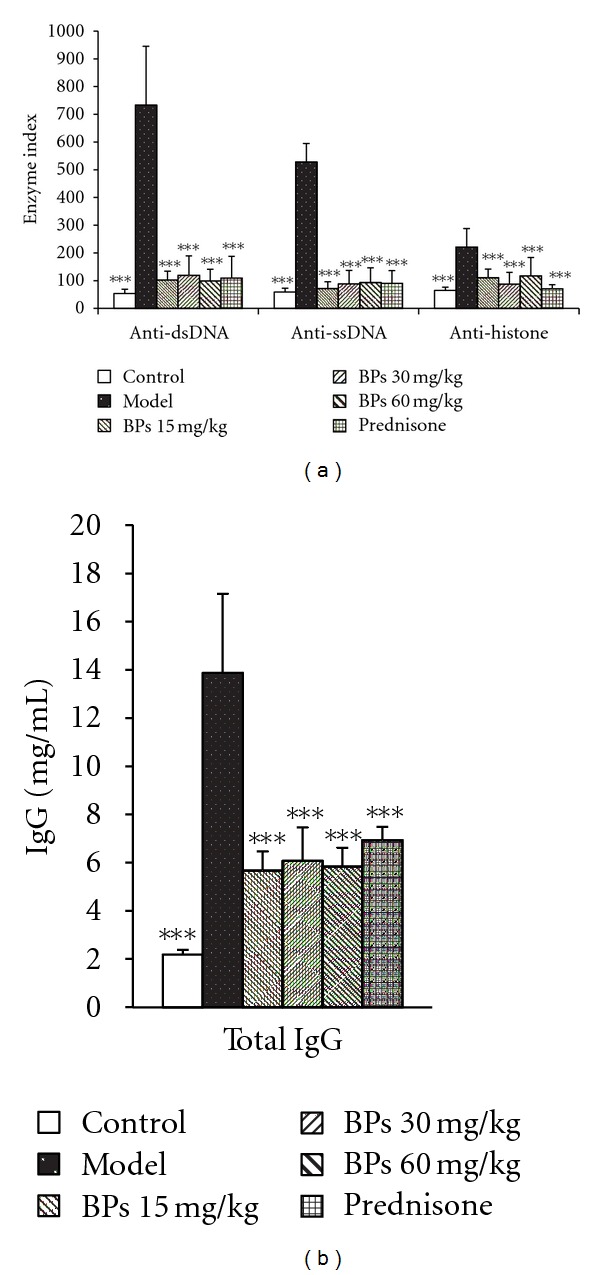
Effect of BPs on antinuclear antibodies and total IgG production in BALB/c and MRL-lpr mice. MRL-lpr mice were grouped randomly and treated with BPs 15, 30, and 60 mg·kg^−1^·day^−1^, prednisone 5 mg·kg^−1^·day^−1^, or model from week 12 to week 24; data were expressed as means ± SD (*n* = 6–8); ***P < 0.001 compared with vehicle-treated model group, tested by ANOVA and Fisher's PLSD. Enzyme Index = 100 × OD_tested_  /(Mean OD_control group_ + 3SD).

The sera were collected from MRL-lpr and BALB/c mice to assay autoantibody concentration by ELISA. Anti-dsDNA, anti-ssDNA, and anti-histone antibody levels were significantly elevated in the vehicle-treated MRL-lpr mice (model group) compared with BALB/c mice (control group) (*P* < 0.001) (Figure 3) and were significantly reduced by BPs 15, 30, 60, or prednisone 5 mg·kg^−1^·day^−1^ (*P* < 0.001). It was indicated that BPs blocked the production of autoantibodies in MRL-lpr mice, which are closely related to SLE.

Furthermore, total IgG levels were assayed. Similarly, total IgG levels were significantly elevated in the vehicle treated MRL-lpr mice compared with control group (*P* < 0.001) (Figure 3) and were reduced by BPs 15, 30, 60, or prednisone 5 mg·kg^−1^·day^−1^ significantly (*P* < 0.001).

### 3.4. BPs Impaired Proteinuria and Reduced Serum Creatinine of MRL-lpr Mice

**Figure 4:**
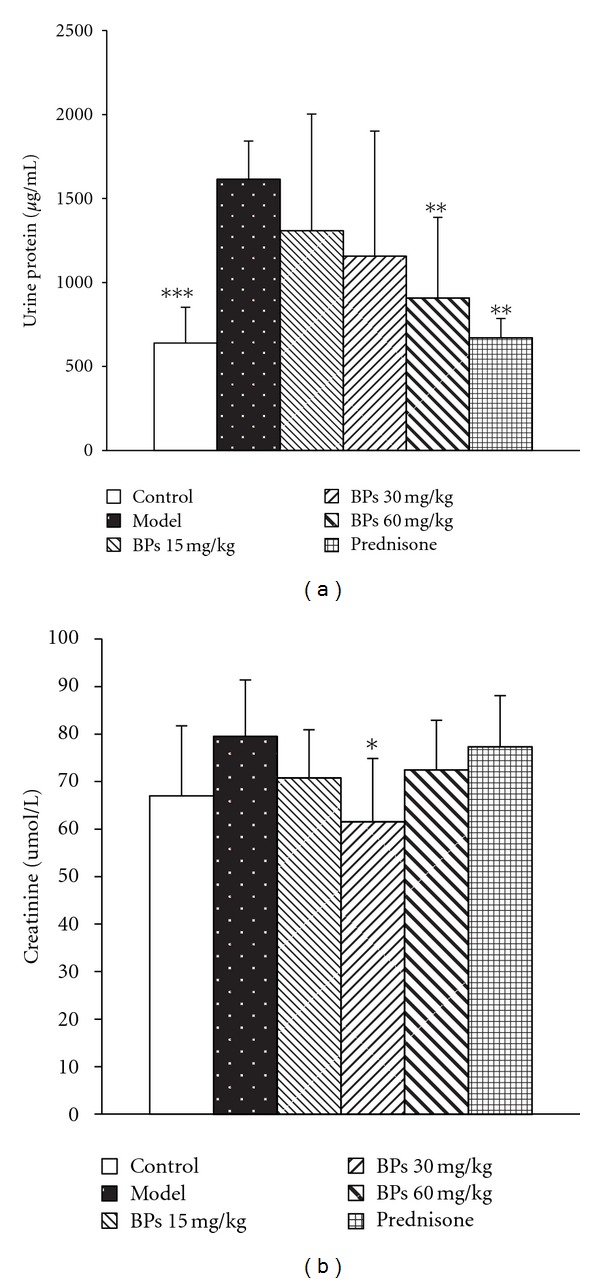
Effect of BPs on urinary protein and serum creatinine levels of MRL-lpr and BALB/c mice. MRL-lpr mice were grouped randomly and treated with BPs 15, 30, and 60 mg·kg^−1^·day^−1^, prednisone 5 mg·kg^−1^·day^−1^, or model from week 12 to week 24; mice were sacrificed and the supernatant of urine was diluted 1 : 3 in normal saline; the serum creatinine was measured with Jaffe method; data were expressed as means ± S.D. (*n* = 6–8); *P < 0.05, **P < 0.01, ***P < 0.001 compared with vehicle-treated model group, tested by ANOVA and Fisher's PLSD.

To evaluate whether BPs had effects on kidney function, MRL-lpr mice were treated by BPs for 12 weeks and urine samples were collected for protein determination. In comparison with control group, model group exhibited significant increase in the level of urinary protein (*P* < 0.001), indicating a certain degree of kidney dysfunction. However, BPs treatment resulted in a reduction in the levels of urinary protein (Figure 4). BPs 60 mg·kg^−1^·day^−1^ and prednisone 5 mg·kg^−1^·day^−1^ significantly reduced urinary protein level (*P* < 0.01).

At 24 weeks of age, MRL-lpr mice showed increased serum creatinine levels (*P* = 0.057), BPs treatment mildly reduced serum creatinine levels, but only 30 mg·kg^−1^·day^−1^ group showed significance (Figure 4).

### 3.5. BPs Suppressed Lupus Nephritis and Reduced Renal IgG Deposition in MRL-lpr Mice

**Figure 5:**
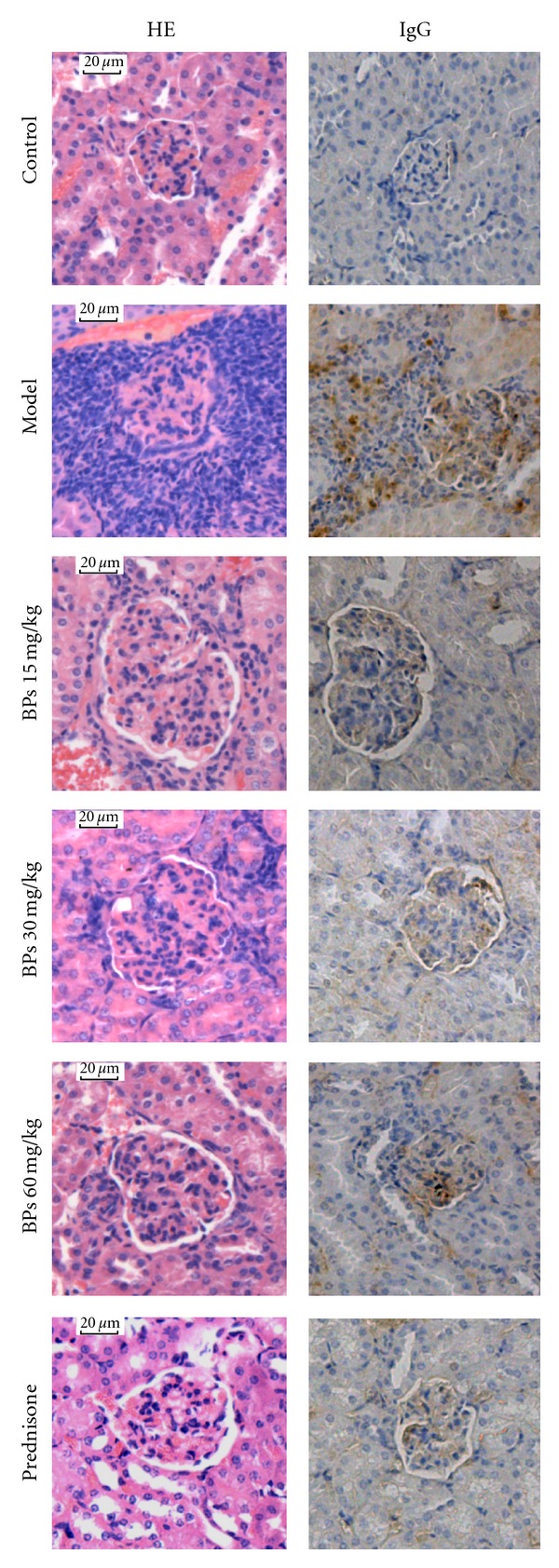
Haematoxylin and eosin-stained and immunohistochemistry of kidney sections. Light microscopy was 200x. Section of a BALB/c group mouse: normal kidney sections (HE, IgG). Section of a vehicle-treated model mouse: glomerular sclerosis, tubular atrophy, and increased infiltrating leukocytes (HE), a patchy dense immunoperoxidase indicative of IgG deposition (IgG). Section from 15, 30, 60 mg·kg^−1^·day^−1^ BP- and prednisone-treated mouse: mild mesangial cell proliferation (HE), less IgG deposition (IgG).

We compared the extent of nephritis between vehicle-treated and BPs-treated MRL-lpr mice at 24 weeks. Vehicle-treated MRL-lpr mice developed diffuse proliferative glomerulonephritis presented as diffuse mesangial matrix expansion, profound mesangial cell proliferation, and focal segmental glomerulosclerosis. Cellular crescents and global glomerulosclerosis were often seen, and the number of both resident cells and infiltrating leukocytes was increased in glomeruli. Most animals revealed profound tubulo interstitial inflammation as characterized by periglomerular and diffuse interstitial leukocyte infiltrates, tubular atrophy, and intraluminal cast formation. Treatment with BPs suppressed lupus nephritis as documented by a significant reduction of the glomerulonephritis scores that encompasses glomerular cell proliferation, endocapillary hypercellularity, crescent formation, sclerosis, tubular atrophy and casts (Table 2, Figure 5).

To determine whether BPs might affect renal disease by reducing IgG deposition in the kidneys, tissue sections from mice were stained for the presence of IgG. Vehicle-treated MRL-lpr mice showed a patchy dense immunoperoxidase indicative of mesangial and tubulointerstitial IgG deposition. In contrast, BPs treatment significantly decreased IgG deposition (Figure 5).

### 3.6. BPs Suppressed Inflammatory Mediators and Markers Expression in Kidney

**Figure 6:**
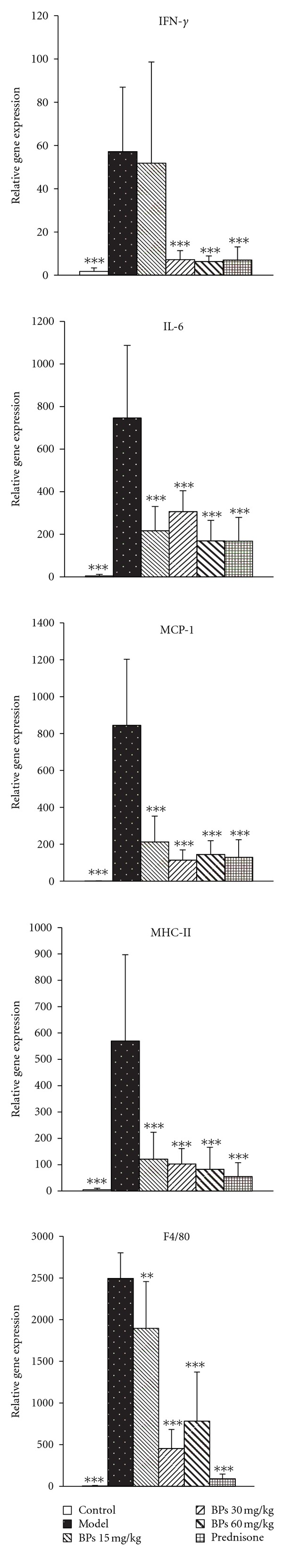
IFN-*γ*, IL-6, MCP-1, MHC-II, and F4/80 mRNA expression in the kidney from MRL-lpr mice. The mRNA expression of IFN-*γ*, IL-6, MCP-1, and MHC-II in the kidney was prepared from the BALB/c mice (control) and MRL-lpr mice administered vehicle solution (model), 15, 30, and 60 mg·kg^−1^·day^−1^ BPs, or 5 mg·kg^−1^·day^−1^ prednisone. The levels of mRNA were analyzed by real-time PCR; GADPH was shown as the loading control. Each data point represented the mean of individual mouse in three independent experiments. Values were presented as means ± S.D. ratio of IFN-*γ*, IL-6, MCP-1, MHC-II, and F4/80 mRNA to GADPH mRNA (*n* = 6). *P < 0.05, **P < 0.01, ***P < 0.001 compared with vehicle-treated model group, tested by ANOVA and Fisher's PLSD.

**Figure 7:**
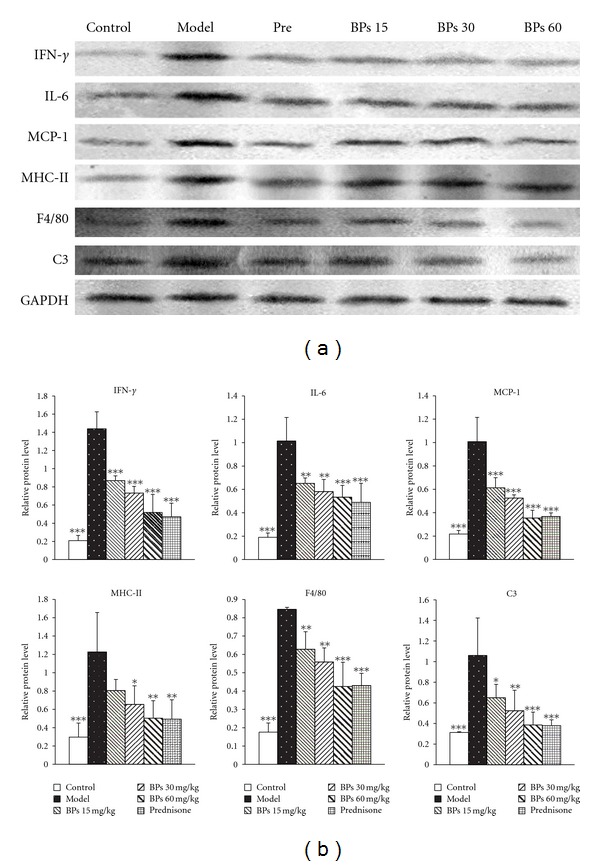
Protein expression of IFN-*γ*, IL-6, MCP-1, MHC-II, F4/80, and C3 in the kidneys of MRL/lpr mice. (a) Renal expressions of IFN-*γ*, IL-6, MCP-1, MHC-II, F4/80, and C3 were examined by Western blot analysis. The presented blot was a representative of those obtained from three mices. GAPDH was used as loading control. (b) Results of quantitative analysis were expressed as means ± S.D. ratio of IFN-*γ*, IL-6, MCP-1, MHC-II, F4/80, and C3 to GADPH (*n* = 3). *P < 0.05, **P < 0.01, ***P < 0.001 compared with vehicle-treated model group, tested by ANOVA and Fisher's PLSD.

Using RT-PCR and Western blotting, we examined the expression of IFN-*γ*, IL-6, MCP-1, MHC-II, and F4/80 in the kidney of mice at 24 weeks. The expression of IFN-*γ*, IL-6, MCP-1 mRNA, and protein was greatly elevated in vehicle-treated MRL-lpr mice and was reduced in varying degree from BP-treated mice compared with the vehicle-treated mice. BPs treatment reduced the mRNA and protein expression of the surface marker MHC-II and F4/80 (Figures 6 and 7).

C3 levels were also reduced in the kidney of BPs treatment group, as was shown in Western blotting results (Figure 7).

## 4. Discussion

Systemic lupus erythematosus is a chronic autoimmune inflammatory disease that affects various organs, including skin, joint, kidney, and blood. Even without clear pathogenesis mechanism, it has been suggested that the main effectors of disease pathology are the diverse autoantibodies, immune complexes, complements, and autoreactive cells. Altered biology of immune cells and possibly other cell types invariably contributes to the progression of the diseases [2].

Macrophages have been considered to play important roles in the pathogenesis of SLE in numerous studies [4]. Owing to the intrinsic defects of macrophages, SLE is one of the widely acknowledged examples in which apoptotic cell clearance is disturbed in both mice and patient [3]. Recent studies also confirmed that aberrant function of lupus macrophages appeared to play a dynamic role in the initiation and perpetuation of the systemic autoimmune response and organ damage [4]. Moreover, aberrant monocyte surface marker expression and numerous abnormalities of the cytokine network have been described in patients suffering from SLE [3].

Our laboratory has done a series of experiments concerning the effect of crude *Bupleurum* polysaccharide (BPs) on the immune system. It was suggested that BPs have potent immunomodulatory activity on macrophages *ex vivo* by enhancing phagocytic activities and inhibiting LPS-induced production of proinflammatory mediators [12]. It had been demonstrated in our previous study that BPs were a major component that contributed to the anticomplementary activity and had a beneficial effect on systemic lupus erythematosus-like syndrome induced by CJ-S_131_ in BALB/c mice [11]. BPs also inhibited LPS-induced phosphorylation of NF-*κ*B, TNF-*α*, IL-1*β*, IL-6, IL-12, and IFN-*β* production in peritoneal macrophages (in press).

Here we find that BPs treatment for 12 weeks has several beneficial effects on MRL-lpr mice including protection from lethality, amelioration of glomerulonephritis, improvement of kidney function, and prevention of lymphadenopathy and thymus enlargement. The improved pathology was associated with reduced production of autoantibodies and inhibited expression of inflammatory cytokines and chemotactic factors in the kidneys. We suggest that these benefitial effects might be related to BPs anticomplement activity and immunomodulatory functions on macrophages. Since analyses on blood, urine, and kidney were performed at the end of the treatment period and the sickest mice in model group had died, the final analyses are likely not an accurate measure of the effects of these agents. More experiments will be needed and the analyses of desease progression should be carried out in our further experiment.

In healthy individuals, the immune system defends the body against microbes by distinguishing self from foreign antigens. For reasons not completely understood, immune tolerance against self-antigens fails in SLE and the immune system actively responds to a wide array of autoantigens [16]. Antinuclear antibodies such as anti-dsDNA antibodies are unique to patient with SLE [17]. One of the most important features of anti-dsDNA antibody is its association with glomerulonephritis [18]. In the present study, the serum from vehicle-treated MRL-lpr mice had higher levels of total IgG, which may contain a large amount of antinuclear antibodies (ANA) mainly. The kidney sections also had higher IgG deposition and expressed hypercellular glomeruli. High levels of protein in urine and creatine in sera also indicated kidney dysfunction in MRL-lpr mice. According to the previous results, it is indicated that the kidney dysfunction in MRL-lpr mice might be related to their aberrant immune responses. We demonstrated that BPs decreased the total IgG, anti-dsDNA, anti-ssDNA, and anti-histone antibodies, as well as renal IgG deposition, reduced glomerular hypercellularity, and suppressed lupus nephritis in MRL-lpr mice. Therefore, it is suggested that BPs improves glomerulonephritis of MRL-lpr mice through modulation of these pathological phenomena.

Lupus nephritis is triggered by glomerular immune complex deposits that activate the components of the classical complement pathway, which finally leads to the assembly of membrane attack complex [19]. In the present study, the favorable effects of BPs treatment on markers of active lupus nephritis were associated with a significant reduction of complement C3 in kidney, a marker of intraglomerular complement activation. This was in line with our previous findings that BPs had anticomplementary activity.

Leukocyte infiltration is a hallmark of severe lupus nephritis, and macrophages play an important role in amplification of the inflammatory process in the kidney [20]. MHC-II is an important molecule expressed on antigen presenting cells, such as macrophages, dendritic cells, and B cells. Enhanced MHC class II antigen expression is a common feature of autoimmunity and may play a key role in the initiation and progression of lupus nephritis [21]. F4/80 is a cell surface glycoprotein predominantly expressed on murine macrophages [22]. In this study, renal RT-PCR and Western blotting revealed that MHC-II and F4/80 were significantly upregulated in the kidney of MRL-lpr mice. This is likely the reflection of aberrant activation and increased infiltration of macrophages in this model [4]. We found that the expressions of MHC-II and F4/80 were significantly inhibited by BPs, which might reflect the immune inhibitory effect of BPs on overactivated macrophages.

MCP-1 can induce transendothelial migration of monocytes, and infiltration of monocytes/macrophages can in turn facilitate tissue destruction [23]. MCP-1 deficient MRL-lpr mice were protected from progressive renal injury by reduced leukocyte recruitment [24]. In this study, BPs treatment resulted in a decrease in renal expression of MCP-1, indicating that the effect of BPs on kidney inflammation might be mediated by reduced MCP-1 expression and macrophage recruitment.

Infiltrating mononuclear cells are the major source of IL-6 in diseased kidneys affected by lupus nephritis [25], IL-6 can in return promote macrophage activation, it is elevated in the serum and urine of some lupus patients, and murine lupus models support a role for IL-6 in nephritis [26, 27]. In our previous study, we found that BPs decreased LPS-induced excessive production of NO and proinflammatory cytokines, including IL-1*β*, IL-6, and TNF-*α*, but had mild effects on these cytokines when they were in physiologic levels [12]. We found that the renal expression of IL-6 was significantly elevated in the kidney of MRL-lpr mice and was inhibited by BPs treatment, indicating the suppression effect of BPs on macrophage secretions.

Tissue macrophages can be recruited and activated as a consequence of the actions of a range of inflammatory mediators such as cytokines. IFN-*γ* is increased in the serum of some SLE patients and its level has been shown to correlate with disease activity [28]. Furthermore, IFN-*γ* is a potent cytokine in inducing MHC-II antigen expression in infiltrating monocytes [29]. We found that the expression of IFN-*γ* was significantly elevated in the kidney of MRL-lpr mice and was inhibited by BPs treatment, indicating that the anti-inflammatory effects of BPs might also be associated with decreased renal expression of proinflammatory cytokines.

In addition to the local suppression of macrophages, the therapeutic effects of BPs might also be related to a systemic blunting of autoimmunity, as reflected by decreased serum levels of autoantibodies and renal immune complex deposition. Since macrophage-derived cytokines are required for the differentiation of B cells into antibody-secreting plasma cells [30] and for the survival and proliferation of B cells [31], one attractive hypothesis could be that the inhibitory effect of BPs on autoantibody production and deposition might be mediated by macrophages. Another plausible hypothesis could be that the effect of BPs treatment is due to less autoantibody deposition in the kidneys with all the other effects being secondary. Although our previous work showed that BPs has an effect on macrophages, and in this study we found a decrease in surface markers and cytokines associated with macrophages (either recruiting them, activating them, or produced by them), further studies are needed to identify whether macrophages are the direct function target of BPs treatment.

In summary, this study demonstrated that BPs improves lupus nephritis mainly by suppressing abnormal autoimmunity of SLE. Our analysis proves the therapeutic efficacy of BPs in the treatment of SLE in MRL-lpr mice. Taken with the current data, BPs could be a new agent for the treatment of autoimmune disease.

## Figures and Tables

**Table 1:** Effect of BPs on lymphadenopathy of MRL-lpr mice.

Weeksof age	lymphadenopathy score				
	Model	BPs			Prednisone
		15 mg·kg^−1^	30 mg·kg^−1^	60 mg·kg^−1^	5 mg·kg^−1^
16	0 (0~1)	0.5 (0~1)	1 (0~1)	0 (0~1)	0 (0~0)
18	1 (0~2)	1.5 (0~2)	1 (0~2)	0 (0~2)	0 (0~1)
20	2 (0~3)	2 (0~3)	1.5 (0~3)	0 (0~3)^a^	1 (0~2)
21	2 (1~3)	2 (0~3)	2 (0~3)	0 (0~3)^a^	1 (0~2)^a^
22	3 (2~3)	2 (0~3)	2 (0~3)	0 (0~3)^b^	1 (0~2)^b^
23	3 (2~3)	3 (0~3)	2.5 (0~3)	0 (0~3)^b^	1 (0~3)^a^
24	3 (2~3)	3 (0~3)	3 (0~3)	0 (0~3)^b^	1.5 (0~3)^a^

note: Data were expressed as median (minimum~maximum) (*n* = 6–8); ^a^ *P* < 0.05, ^b^ *P* < 0.01 compared with vehicle treated model group, tested by Mann-Whitney *U* test.

**Table 2:** Effect of BPs on glomerulonephritis scores of BALB/c and MRL-lpr mice.

			Glomerulonephritis score		
Control	Model	BPs			Prednisone
		15 mg·kg^−1^	30 mg·kg^−1^	60 mg·kg^−1^	5 mg·kg^−1^
0(0~1)^c^	3.5 (3~4)	2 (1~3)^c^	2 (1~2)^c^	2 (1~2)^c^	1.5 (1~2)^c^

note: Data were expressed as median (minimum~maximum) (*n* = 6–8); ^b^ *P* < 0.001 compared with vehicle-treated model group, tested by Mann-Whitney *U* test.
